# As predicted by hyperfunction theory, rapamycin treatment during development extends lifespan

**DOI:** 10.18632/aging.203937

**Published:** 2022-03-05

**Authors:** Mikhail V. Blagosklonny

**Affiliations:** 1 Roswell Park Comprehensive Cancer Center, Buffalo, NY 14263, USA

**Keywords:** quasi-programmed aging, mTOR, sirolimus, growth, reprogramming

As suggested in 2006, by slowing down the mTOR-driven developmental program, rapamycin must slow down quasi-programmed aging [1]. In other words, targeting development with rapamycin must lead to a longer lifespan. An elegant study by Gladyshev and co-workers has confirmed this prediction [2].

According to hyper-function theory, aging is a quasi-program, a purposeless continuation of the growth program that has not been switched off upon its completion [1]. Aging is not programmed, only development is. Unlike a program, a quasi-program has no aim, although, like a program, it can be modulated [1,3,4]. For example, excessive nutrients and calorie restriction can accelerate and decelerate aging, respectively.

Aging is driven by hyperfunctional signal-transduction pathways which, via cellular and systemic hyperfunctions, cause age-related diseases, whose sum is called aging [1]. Hyperfunctions cause organ damage (not molecular damage), resulting in loss of functions and secondary functional decline [1,5].

The nutrient-sensing mTOR pathway promotes cellular growth [6–8] and cellular senescence, which is a continuation of cellular growth, when the cell cycle is blocked [9,10]. According to hyperfunction theory, age-related diseases are quasi-programmed [1,11] with clear-cut examples in simple organisms such as *C. elegans* [11–15]. Hyperfunction theory was extensively reviewed [1, 5, 11, 16 -20]. Critical comments [21–23] have been addressed [5, 24]. Importantly, hyperfunction theory is mTOR-centric, describing mTOR-driven aging and its diseases [1]. By slowing down aging, rapamycin delays age-related diseases [1,25,26].

To maximally extend health and lifespan in humans, it was suggested that the treatment with rapamycin should be started at a young age: “As an anti-aging drug, rapamycin will prevent diseases rather than cure complications of diseases. Rapamycin will prevent [organ] damage but not to reverse damage. It might prevent diabetes and obesity but not diabetic gangrene and stroke. It might prevent macular degeneration but will unlikely cure blindness. Rapamycin will not repair broken bones but might prevent osteoporosis… rapamycin will be most useful as [an] anti-aging drug to slow down senescence and to prevent diseases” [1].

It was suggested in 2006 to take rapamycin immediately to the clinic to suppress human aging [1], even though longevity studies in animals were not yet performed. Starting from 2009, numerous studies demonstrated that rapamycin extends lifespan in mice [27–39].

Hyperfunction theory predicts that rapamycin can slow down aging by two complementary mechanisms:

(a) directly suppressing the quasi-program of aging

b) reprogramming aging by slowing the developmental-growth program

To demonstrate reprogramming, rapamycin should be given for a brief period during development.

Shindyapina et al. showed that treatment with rapamycin for the first 45 days of life extends median lifespan by 10% [2]. Health was improved as measured by gait speed, frailty index, and glucose and insulin tolerance tests [2]. Rapamycin-treated mice were small and did not catch up on growth later [2].

The hyperfuntion theory explains why a large-body correlates with longevity between species (for example, elephants live longer than mice, which live longer than flies), but in contrast, within each species, it is a small body size that is associated with longevity [40]. Life-long small body size after a brief treatment is consistent with reprogramming of the growth program.

Notably, life extension by rapamycin was mostly observed in male mice [2]. This is consistent with the finding that mTOR is overactivated in young male mice compared with young female mice, thus explaining robustness of males at young age and their shorter lifespan [41].

Supporting the notion of rapamycin-induced reprogramming, previous studies found that (a) even transient treatment with rapamycin can extend lifespan [27,36,39] (b) a single rapamycin injection can lower body weight set point in the long run [42] and (c) rapamycin can affect the mTOR pathway activity long term by preventing obesity [43,44].

## Further suggestions

To further study rapamycin-induced reprogramming of aging, pregnant mice should be treated with a single subcutaneous injection of rapamycin and the lifespan of their offspring should be measured. Prenatal (before birth) rapamycin treatment on early postnatal development has been studied [45–47]. For example, prenatally rapamycin-treated neonates are small, and body weight and left ventricular mass remain reduced in adulthood [47]. However, lifespan was not measured. (Note: rapamycin pre-treatment increased mortality immediately after the birth [47] because mTOR is essential early in life. Early-life death is not aging-driven and should be excluded from the age-related mortality curve).

At what age may rapamycin treatment be started in order to maximally extend human lifespan? Based on murine data, treatment with rapamycin can be started at a very old age. Still, in theory, the maximal effect potentially may be achieved before age-related diseases and pre-diseases become apparent in humans [1]. However, it should not be started too early because mTOR is essential for growth and early life fitness. In my opinion, rapamycin treatment (for anti-aging purposes) may only be started when a young adult can make informed decisions and should not be allowed before the age of 21. Doctors should consider that rapamycin may negatively affect reproduction, albeit reversibly. I believe that the initial dose should be very low and gradually increase with older age, when full individual doses are achieved. An anti-aging dose/schedule is a maximum dose that do not yet cause side effects in a particular person [48]. Self-treatment is unacceptable and doses are highly individual [48,49].

## Disclaimer

This commentary is for information purposes, not medical advice.
